# Bacterial microbiota associated with raw plant-based meat analogue products and their influences on selective enrichment for *Escherichia coli* O157:H7

**DOI:** 10.1016/j.crfs.2024.100944

**Published:** 2024-12-01

**Authors:** Sabrina Capitani, Liam P. Brown, Catherine D. Carrillo, Calvin Ho-Fung Lau

**Affiliations:** Ottawa Laboratory (Carling), Canadian Food Inspection Agency, Ottawa, Ontario, Canada

**Keywords:** Plant-based meat analogues, Alternative protein, Microbiome, Bacterial community, Cultural enrichment, *E. coli* O157:H7, 16S rRNA gene sequencing

## Abstract

Towards fostering a more sustainable food production system in face of the climate change challenge, alternative protein meat-substitute products that are plant-based and free of animal by-products have been gaining attractions from both food manufacturers and consumers. With these so-called plant-based meat analogues (PBMAs) becoming increasingly available at supermarkets, there is very little known about their microbial properties. In this short report, we characterized the bacterial composition of raw plant-based ground meat imitation retail products using 16S rRNA gene amplicon sequencing. Despite the observed bacterial community dissimilarity between sample brands, a total of 18 shared genera (dominated by Bacilli and Gammaproteobacteria classes) were identified as the core constituents of the bacterial microbiota of these PBMA products. Within the scope of food safety testing, to gain insights on the dynamics of the enrichment process for *E. coli* O157:H7 in accordance with the Health Canada reference method MFHPB-10, bacterial taxonomic analyses were conducted at different stages of the prescribed cultural procedures. Using both control and *E. coli* O157:H7-inoculated PBMA samples it was revealed that, independent of the presence of *E. coli* O157:H7, off-target bacteria of the *Clostridium sensu stricto 1* genus were significantly enriched from the uncultured samples. Additionally, the abundance of *Hafnia-Obesumbacterium* bacteria in the PBMA samples was also increased in the enrichment products, but only when *E. coli* O157:H7 was absent. Consistent with the spread-plating results indicating that the inoculated *E. coli* O157:H7 cells were capable of reaching a high density (>10^8^ CFU/ml) in the resultant enrichment cultures, the significant enrichment of bacterial 16S rRNA gene sequences belonging to the targeted genus of *Escherichia,* but not *Hafnia-Obesumbacterium*. This further highlights the competitive nature of the selective enrichment for *E. coli* O157:H7 against specific background bacteria associated with the PBMA products.

## Highlights

• The bacterial composition of raw plant-based ground meat imitation products were described using 16S rRNA sequencing.
• A total of 18 genera were identified as the core constituents of these alternative protein products' bacterial microbiota.
• The cultural dynamics of *E. coli* O157:H7 and product-associated bacteria was examined under a 2-step enrichment procedure.
• Apart from the O157:H7, significant co-enrichment of off-target bacteria including *Clostridium sensu stricto 1* was detected.

## Graphical abstract

**Figure:**
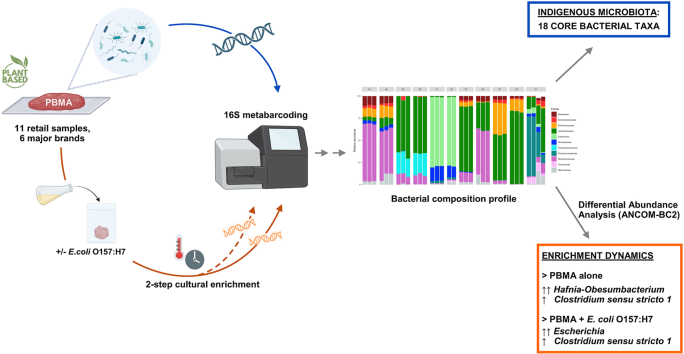

## 1 Introduction

With the growing awareness and understanding about climate change and gut health, there has been a movement of transforming our food production system to become more sustainable and less dependent on industrialized livestock farming. Increasing popularity of vegetarian or vegan diets for health and ethical reasons will result in increased demand for meat replacement products that are plant-based and free of animal derivatives. In 2023, the global market size of plant-based meat-substitute products was valued at USD 7.17 billion, with a forecasted growth rate of approx. 19.4% per annum up to 2030 (Grand View Research, 2024). To meet the sensory expectation and gastronomic preference of general consumers, food companies have recently developed a second generation of plant-based meat analogue (PMBA) products that resemble the appearance, taste, texture and nutritional profile (protein, fat) of their counterparts made out of animal by-products (Jang and Lee, 2024). Unlike other fabricated vegetarian meat replacements such as tofu, tempeh, Seitan, and Quorn, these newer PBMA products are primarily composed of protein derived from pea and/or soy and non-animal fat (coconut, sunflower, avocado and/or canola oil), together with a plethora of seasoning and flavoring supplements and novel additives (Jang and Lee, 2024; Singh et al., 2021). As such, not only are they classified as highly-processed foods (Boukid, 2021), they may also harbour significant foodborne pathogen(s) due to increased potential for microbiological contamination through compromised raw ingredients and/or extensive manufacturing procedures. Additionally, given the habitual behavior of consumers towards handling and cooking certain animal meat products (e.g. minced meat, hamburgers), it is reasonable to assume that the raw PBMA products share some of the same food safety concerns as the meat products they are mimicking. Although there have yet to be any reported cases of foodborne illness associated with these plant-based meat-substitute products, at the time of writing, a serious outbreak of *Listeria* infections linked to plant-based refrigerated beverages resulting in at least 15 people being hospitalized and 3 death across Canada is currently under active investigation (Public Health Agency of Canada, 2024). Given the uniqueness of this alternative protein food, little is known about the microbial properties of raw PBMA products and their ecological significance during food inspection activities aimed at detecting pathogenic bacterial contaminants.

*Escherichia coli* O157:H7 is a prototypic Shiga-toxin producing strain of *E. coli* (STEC) that can cause hemorrhagic colitis and severe hemolytic uremic syndrome in human (Baker et al., 2016). Since its first identification in 1982 from undercooked hamburger meat (Riley et al., 1983), *E.coli* O157:H7 has been repeatedly implicated in significant foodborne illness outbreaks involving ground meat, dairy products, fresh produce, and ready-to-eat foods. Although STEC O157:H7 is most frequently associated with foods of animal origin, (in-) direct cross-contamination with this zoonotic pathogen can occur at any point along the farm-to-fork continuum for fresh and processed food alike. To mitigate the risks associated with foodborne STEC, culture-based isolation and identification of *E. coli* O157:H7 has been one of the most definitive diagnostic approaches to be applied in food safety monitoring and investigation scenarios. In principle, to enable the sensitive recovery of a bacterial target that could have a low-level presence in food products, samples are first subjected to a single or serial controlled laboratory condition(s) for the purpose of culturally enriching for a specific organism in order to attain its minimal detectable level. A key determinant that can significantly influence the outcome of an O157:H7-targeting cultural enrichment procedure is the sample-associated microbiota. Depending on the culturing conditions, some of these background bacteria can also be selected for (i.e. enriched), with or without antagonistically competing for space and resources against the intended microbial target. With the objectives of promoting STEC recovery and suppressing the overgrowth of non-STEC background, a common cultural strategy shared by a number of current standard methods for the detection of *E. coli* O157:H7, including ISO 16654:2001 (International Organization for Standardization, 2001), MLG 5C.03 (United States Department of Agriculture, 2023) and MFHPB-10 (Health Canada, 2017), involves overnight incubation of sample homogenized in modified Tryptone Soya Broth (mTSB) or mTSB supplemented with novobiocin (mTSB-n).

In the present study, we aimed to profile and characterize the bacterial microbiota of plant-based ground meat imitation products of different major PBMA brands using 16S rRNA gene amplicon sequencing. By simulating *E. coli* O157:H7 contamination scenarios, the cultural dynamics of this significant foodborne pathogen and any background bacteria associated with the raw PBMA products was systemically examined under the mTSB-based enrichment conditions as prescribed in the Canadian *E. coli* O157:H7 method MFHPB-10.

## 2 Methods and materials

**Bacteria and culture condition***Escherichia coli* strain OLC0795, a nalixidic acid-resistant derivative of the verotoxigenic *E. coli* O157:H7 strain ATCC 35150 (Blais et al., 2008), was grown from single colonies in Brain Heart Infusion (BHI) medium (Oxoid, Nepean, ON, Canada) for 16−18 h at 37^o^C with mild agitation in a shaker incubator. Ten-fold serial dilutions of OLC0795 culture were prepared in BHI medium and were used in sample inoculation as described below. To confirm the approximate number of *E. coli* O157:H7 cells being introduced into the food samples, 100 μL of the diluted inocula was plated, in triplicate, onto BHI agar and incubated at 37^o^C for 18−20 h before enumeration.

**Sample description and processing** A total of eleven samples belonging to six different brands of raw plant-based meat analogue (PBMA) products that are labelled as “simulated ground” and “contains no meat” were acquired over the period of Nov 2021 to July 2022 from grocery stores in Ottawa, Ontario, Canada. All samples were purchased in the form of vacuum-sealed or modified/controlled atmosphere packaging available in the refrigerated goods section. Typically, samples were maintained and stored in their original packaging at 4^o^C for no more than 24 h after purchasing and were subsequently processed by following either Workflow 1 alone or both Workflows 1 and 2 as illustrated in Fig. S1. *Workflow 1*: To characterize the bacterial microbiota of the raw PBMA product samples, 25-g portions of samples were aseptically transferred to individual 55 oz. Whirl-pack® sampling stomacher bags (Thermo Fisher Scientific) in triplicate. Each of the bagged samples was then homogenized in 225 mL of modified Tryptone Soya Broth (mTSB), followed by blending for 1 min at a speed setting “1” in a Bag-Mixer ® 400VW (Interscience Laboratories, Woburn, MA, United States). After sampling of the homogenate filtrate (i.e. non-enriched), stomacher bags containing the homogenized PBMA samples were incubated at 37^o^C for 24 h, before further collecting samples of the resultant bacterial cultures (i.e. enriched). *Workflow 2*: To simulate *E. coli* O157:H7 contamination incidents, additional stomacher bags containing 25 g of a representative plant-based ground sample for each of the six PBMA product brands were inoculated with 1 mL of *E. coli* strain OLC0795 inocula of different concentrations, targeting ≤1 log_10_ CFU (low-level) and 3 log_10_ CFU (high-level) per unit of sample (Table S1). Inoculation-free controls were included by introducing 1 mL of the BHI medium diluent into the sample-containing bags. Both inoculated and non-inoculated control samples were prepared in duplicate. After massaging the sample-containing stomacher bags manually to facilitate the homogenous distribution of the inoculum, they were left at 4^o^C for 24 h before selective cultural enrichment.

**Selective cultural enrichment and detection of *E. coli* O157:H7 from raw PBMA products** Based on the MFHPB-10 method listed under the *Compendium of Analytical Methods* of Health Canada (Health Canada, 2017), both artificially-contaminated and inoculation-free control samples were subjected to a two-step cultural enrichment procedure, with simultaneous collections of time-stamped enrichment samples for bacterial community analyses. Briefly, each sample was homogenized in 225 mL of mTSB medium supplemented with 20 mg/L of novobiocin (mTSB-n) through manual massaging of the stomacher bag, followed by blending in a Bag-Mixer ® 400VW for 1 min, and then incubating at 42^o^C for 24 h to obtain the primary enrichment. To obtain the secondary enrichment, 1 mL of primary enrichment culture was transferred into 9 mL of Enterohemorrhagic *E. coli* Enrichment Broth (EEB), which is composed of mTSB with the inclusion of 8 mg/L of vancomycin, 10 mg/L of cefsulodin and 50 ng/L of cefixime, and incubated at 35^o^C for an additional 24 h. Apart from collecting post-enrichment samples from the respective primary- and secondary-enrichment cultures, ten-fold serial dilutions of the primary cultures were also plated onto the selective chromogenic Rainbow® Agar O157 (Biolog, Hayward, CA, United States) in duplicate as an MFHPB-10 alternative method to infer the extent of *E. coli* O157:H7 recovery. As per manufacturer's instructions, and using the *E. coli* O157:H7 inoculum as reference, colonies with grayish-black color were presumptively identified as *E. coli* O157:H7. To estimate the total population size of the indigenous bacterial community from the PBMA products examined, ten-fold serial dilutions of the non-enriched sample homogenates recovered from the inoculation-free samples were plated onto the 3M™ Petrifilm™ Aerobic Count Plate in replicates. Unless otherwise specified, both Rainbow® Agar O157 and the 3M™ Petrifilm™ were incubated at 37^o^C for 24−48 h before enumeration.

**Microbiota and bacterial DNA extraction** To recover the total bacterial content from the pre- and post-enrichment samples of individual PBMA products, homogenate filtrates/cultures were centrifuged at 500×*g* for 5 min at 4 °C immediately after their collection. With the exclusion of any precipitated non-microbial particle, the supernatant was then further centrifuged at 13,000×*g* for 20 min to pellet the bacteria. After discarding the supernatant and an additional round of centrifugation at 13,000×*g* for 5 min, any residual liquid was further removed from the tube and the resultant pellet stored at −20 °C. Bacterial genomic DNA was isolated from thawed pellets using the DNeasy™ PowerSoil Pro Kit (Qiagen, Mississauga, ON, Canada) and a Bead Mill-24 homogenizer (Thermo Fisher Scientific) according to the manufacturer's instructions. Sample DNA quantitation was routinely performed using the fluorescence-based Qubit™ dsDNA HS Assay Kit (Thermo Fisher Scientific).

**Bacterial profiling 16S rRNA gene sequencing** To determine the composition of the bacterial communities recovered from PBMA samples and their corresponding enrichment cultures, an Illumina 16S metagenomics sequencing workflow (Illumina Canada, Vancouver, BC, Canada) targeting the V3 and V4 variable region of the 16S rRNA gene was employed (Illumina, 2013). Briefly, the V3 and V4 region of the 16S rRNA gene (∼460 bp) was amplified from the sample DNA in a 25 μl PCR mixture containing 12.5 ng of template DNA, 200 nM of each of the 16S amplicon PCR forward and reverse primers, and 1 x KAPA HiFi HotStart ReadyMix (Roche, Millipore-Sigma, Mississauga, ON). The PCR mixtures were heated for 3 min at 95^o^C, followed by 25 cycles of 30 s at 95 °C, 30 s at 55 °C, 30 s at 72 °C, before finishing with 5 min at 72 °C. The 16S rRNA gene amplicons were purified using AMPure XP magnetic beads (Beckman Coulter, Mississauga, ON, Canada), and adaptor-incorporated unique dual-indices were added to the amplicons using the Nextera XT index Kit v2 (Illumina, Canada) in accordance with the Illumina protocol. Indexed amplicon libraries were then purified and normalized using the NGS normalization 96-well kit (Norgen Biotek, Thorold, ON, Canada) before pooling to a final concentration of 4 nM. High-throughput sequencing was performed on a MiSeq sequencer (Illumina, Canada) using the MiSeq v3 kit (600-cycle) and a loading concentration of 8 pM with 10% PhiX spike-in, to generate 2 x 300 bp paired-end sequences, targeting an output of 100,000 raw reads/sample.

**Bioinformatics analyses** The Nextflow-based nf-core/ampliseq workflow v2.7.1 (Straub et al., 2020) was used for downstream processing of the 16S rRNA amplicon sequencing output. First, sequence data quality was assessed using FastQC (Andrews, 2010) and MultiQC (Ewels et al., 2016). Next, primer sequences were removed from the 5′-ends of sequences with Cutadapt (Martin, 2011), and forward and reverse reads were truncated at positions 277 and 219 bp, respectively, to remove low-quality bases at the 3′-ends. After sequence trimming, DADA2 was employed to remove PhiX contamination, discard reads with >2 expected errors, correct remaining errors, merge read pairs, remove PCR chimeric sequences, and to infer exact amplicon sequence variants (ASVs). To assign taxonomy to the resulting ASVs, SILVA prokaryotic SSU reference database (release 138.1) was used for training the DADA2 sequence classifier. Finally, the ASV sequences and their corresponding taxonomies and abundances were loaded into QIIME2 (Bolyen et al., 2019) to remove ASVs corresponding to mitochondria and chloroplast DNA.

**Statistical and data analysis** Unless otherwise specified, microbial compositional analysis was conducted using R (ver. 4.2.2) and RStudio (ver. 1.4.1106) (R Core Team, 2021). The DADA2-derived taxonomic assignment outputs were processed using microbiome analytical packages *qiime2R* ver. 0.99.6 (Bisanz, 2018) and *phyloseq* ver. 1.42.0 (McMurdie and Holmes, 2013) implemented in R. Visualizations were generated using the R packages *ggplot2* ver. 3.4.1 (Wickham, 2016) and *UpSetR* ver. 1.4.0 (Conway et al., 2017). Contaminant sequences from negative control samples were identified and removed using the “prevalence method” of the R *decontam* package (Davis et al., 2018). To estimate taxonomic alpha diversity, observed richness was computed at genus-level using R package *vegan* ver. 2.6–4 (Oksanen et al., 2020). Beta diversity was estimated by performing principal coordinate analysis (PCoA) based on the weighted Bray-Curtis dissimilarity using the “vegdist” and “pcoa” functions of R packages *vegan* and *ape* ver. 5.6–2 (Paradis and Schliep, 2019). Significance of dissimilarity between groups were examined by permutational multivariate analysis of variance (PERMANOVA) using *vegan* function “adonis2” with 999 permutations. The differential abundances of individual taxa between groups associated with different enrichments stages were determined using the Analysis of Compositions of Microbiomes with Bias Correction (ANCOM-BC2) methodology (Lin and Peddada, 2020), conducted by running the “ancombc2” function of R package *ANCOMBC* ver. 2.02 with default options to take into account the independent variables of enrichment medium and brand as fixed-effects. Any differential abundance with statistical significance at the bacterial taxonomic ranks of genus based on the mixed-directional false discovery rate-controlled Dunnett's type of test output of ANCOM-BC2 were reported in log-scale (natural log) as log fold changes (with standard errors) relative to the pre-enrichment reference.

**Data availability** All biological sequence data are accessible on the NCBI server under BioProject identifier PRJNA1158801. Nucleotide sequences for 16S rRNA amplicon sequence data were submitted to the Sequence Read Archive (SRA) under SAMN43549675–SAMN43549822.

## 3 Results and discussion

**Fig. 1:**
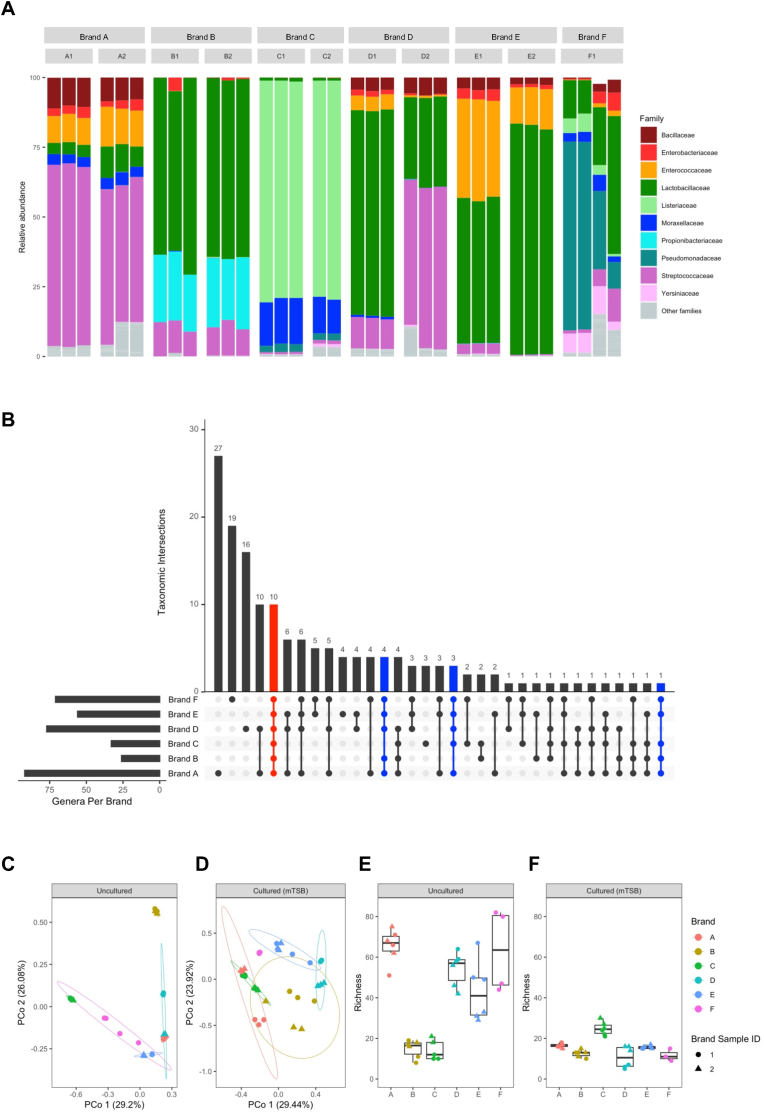
**Bacterial profile of raw plant-based ground meat simulation products. A**, Family-level composition of plant-based meat analogues (PBMAs)-associated bacteria. Each stacked bar represents an independent sample replicate. **B**, Number of shared and unique bacterial genus among samples belonging to different PBMA brands depicted in an *UpSet* plot. The bar chart (*top*) illustrates number of non-redundant genera detected from intersecting brand(s) highlighted using filled-in cells (*bottom*). **C-F**, Principal coordinate analysis of weighted Bray-Curtis dissimilarity matrix (*C, D*) and taxa richness at the genus-level (*E, F*) of bacterial communities recovered from uncultured (*C, E*) and cultured (*D, F*) retail PBMA samples. Each dot represents a replicate of individual PBMA samples colored according to brand. The percentages of total variance explained by each principal coordinate are displayed in the axis labels (*C, D*).

Based on the bacterial profiling results obtained using 16S rRNA gene metabarcoding, it was determined that the microbiota associated with raw, ground meat-mimicking PBMA products are typically dominated by Bacilli (class) bacteria including those belonging to the families of *Lactobacillaceae, Streptococcaceae* and *Enterococcaceae* (Fig. 1A). Without considering those with lone occurrence in the entire sequencing dataset (i.e. singleton taxa)*,* a total of 154 bacterial genera were identified from the eleven individual samples and their corresponding enrichment cultures, representing six different brands of plant-based meat-substitute product. While some of the observed genera appeared to be only associated with products of a specific brand − ranging from a count of 27 (*Brand “A”*) to zero (*Brand “B”*), ten distinct genera could be universally detected across all sample brands, with an additional eight genera frequently detected in five of the six PBMA brands (Fig. 1B–Table 1). When excluding 16S reads assigned to taxa representing typical constituents of spoilage microbiota (namely, *Brochothrix* and *Psychrobacter*), these 18 shared bacterial genera collectively account for a mean of ∼80% of the remaining reads obtained directly from individual PBMA samples without culturing (Fig. S2). As such, they were defined as the core members of the indigenous bacterial microbiota of these uncooked plant-based products. In terms of microbial community (dis)similarity, principal coordinates analyses revealed significant clustering of both natural (Fig. 1C) and cultured PBMA samples (Fig. 1D) (PERMANOVA *p* = 0.001, Table S2), indicating that most of the bacterial community composition variance (85.3% and 62.2%, respectively) could be attributed to brand differences. It is worth noting that despite their substantially higher microbial contents estimated by total aerobic plate count (Table S3), the bacterial richness observed from the uncultured “Brand C″ products was among the lowest (Fig. 1E). As well, in contrast to the expected loss in taxon richness caused by culture-based selection, more unique genera were identified from the cultured versus un-cultured “Brand C″ samples (Fig. 1E and F). These observations imply that the overall bacterial diversity for the natural microbiota of “Brand C″ samples might be somewhat under-estimated, which could potentially be linked to the overwhelming abundance of *Brochothrix* bacteria in the acquired samples of this particular brand (Fig. S2).

**Table 1:** Core bacterial constituents of raw plant-based meat analogue (PBMA) products examined in this study.

Phylum	Class	Family	Genus
Firmicutes	Clostridia	*Clostridiaceae*	*Clostridium sensu stricto 1*

	Bacilli	*Bacillaceae*	*Bacillus*
		*Enterococcaceae*	*Enterococcu*
		*Streptococcaceae*	*Streptococcus*
			*Lactococcus*
		*Lactobacillaceae*	*Lactobacillus*
			*Latilactobacillus*
			*Leuconostoc*
			*Levilactobacillus*
			*Limosilactobacillus*

Proteobacteria	Gammaproteobacteria	*Hafniaceae*	*Hafnia-Obesumbacterium*
		*Moraxellaceae*	*Acinetobacter*
		*Pseudomonadaceae*	*Pseudomonas*
		*Yersiniaceae*	*Serratia*
		*Enterobacteriaceae*	*Enterobacter*
			*Escherichia-Shigella*
			*Klebsiella*
			*Weissella*

From the perspective of foodborne *E. coli* O157:H7 diagnostic testing, to characterize the population dynamics of bacterial community associated with raw PBMA products undergoing a standard two-step cultural procedure prescribed in the MFHPB-10 method prior to the downstream isolation/detection of this significant pathogen (Health Canada, 2017), cultures derived from both control and O157:H7-contaminated PBMA samples were collected at different enrichment stages, followed by culture-dependent recovery of O157:H7 bacteria and culture-independent 16S bacterial community profiling. Of the five representative PBMA samples covering all brands except “Brand E″, presumptive *E.coli* O157:H7 bacteria could be readily recovered from both the primary (mTSB-n) and the secondary (EEB) enrichment cultures of every inoculated sample using the chromogenic Rainbow® Agar O157, but not from those of the inoculation-free controls. For the “Brand E″ sample (E2), due to the presence of atypical *Hafnia* bacteria with confirmed false-positive colony phenotype on the chosen plating medium (unpublished data) which has also been reported elsewhere (Manafi and Kremsmaier, 2001), the attempt to culturally evaluate the recovery of O157:H7 bacteria from products of this brand was prohibited. According to the bacterial counts obtained from the primary enrichment cultures (Table 2), it appeared that 24 h of culturing in mTSB-n was effective in promoting the growth of the *E. coli* O157:H7 target to attain high density, and that the ca. 100-fold difference in the target inoculation level had little, yet-significant (paired *t*-test, *p = 0.03*), influence on the final enriched O157:H7 population size. Consistent with the notion that mTSB-n can provide for the necessary selective pressure against the PBMA-associated bacteria in favor of the O157: H7 target proliferation, 16S rRNA sequencing results revealed average values of 89.5 ± 13.0% (n = 28) and 0.15 ± 0.36% (n = 17) for the relative proportions of presumed *Escherichia* bacteria in the primary enrichment cultures derived from the O157:H7-contaminated and control PBMA samples, respectively (Table S4). The significant enrichment of *Escherichia-Shigella* 16S rRNA sequences in only the contaminated samples, but not in the inoculation-free controls, is also a direct outcome of the mTSB-n –mediated cultural enrichment of the *E.coli* O157:H7 bacterial contaminant specifically.

**Table 2:** Recovery of *E. coli* O157:H7 from artificially-contaminated plant-based meat analogue (PBMA) samples through cultural enrichment.

Sample ID	Presumptive *E.coli* O157:H7 abundance level after primary enrichment (x 10^8^ CFU/ml) a^,^^b^^,^^c^		
	Control	Low	High
A2	0 ± 0	11 ± 3	14 ± 2
B1	0 ± 0	15 ± 4	16 ± 6
C1	0 ± 0	9 ± 2	13 ± 2
D1	0 ± 0	13 ± 1	19 ± 9
E2	NA	NA	NA
F1	0 ± 0	19 ± 6	20 ± 9

a Samples were pre-inoculated with a model *E.coli* O157:H7 strain at the indicated levels. Control, no inoculation; Low, ∼14 CFU/25 g; High, ∼1200 CFU/25 g.

b Numbers represent mean ± standard deviation values obtained from at least three independent determinations for each sample replicate and by spread-plating serial-dilutions of individual enrichment cultures onto Rainbow® Agar O157.

c NA, not available. Sample E2 carried non-targeted *Hafnia* bacteria and resulted in false-positive results for both inoculation-free controls and inoculated samples.

**Fig. 2:**
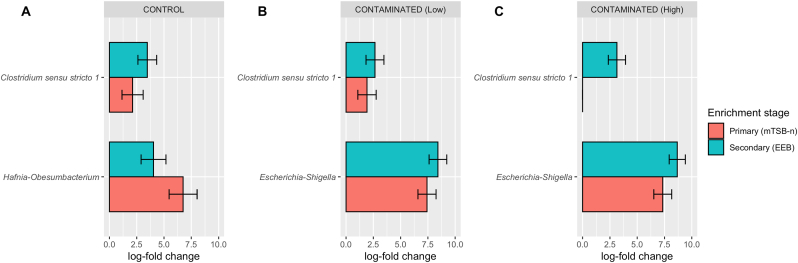
**Selective enrichment of plant-based meat analogues (PBMAs)-associated bacteria from control (*A*) and *E. coli* O157:H7*-*contaminated (*B, C*) samples throughout a two-step cultural procedure.** Retail PBMA samples (*n =* 6) pre-inoculated with different levels of *E. coli* O157:H7 in duplication were subjected to bacterial enrichment by first culturing in modified Tryptone Soya Broth supplemented with novobiocin (mTSB-n) at 42^o^C for 24 h, followed by passaging the primary enrichment products into Enterohemorrhagic *E. coli* Enrichment Broth (EEB) with further incubation at 35^o^C for 24 h. Differential abundance analysis ANCOM-BC2 was conducted to identify bacterial genera that were significantly enriched after the primary (*red,* mTSB-n) and secondary (*blue*, EEB) culturing. Horizontal bars represent, in log-scale (natural log), fold change ± standard error (shown as error bars) of absolute abundance associated with the primary- and secondary-enrichment cultures relative to the pre-enriched reference samples. Only taxa with statistically significant change in their abundance relative to the reference group were displayed.

Given the compositional nature of microbiome 16S rRNA amplicon data (Gloor et al., 2017), bias-corrected differential abundance analyses were performed to identify bacterial taxa whose abundances were increased by the primary and secondary enrichment procedures (Fig. 2). Among the PBMA samples that were free from exposure to any *E. coli* inocula, bacterial taxa assigned to the core-genera *Clostridium sensu stricto 1* and *Hafnia-Obesumbacterium* (Table 1) were significantly more abundant (i.e. enriched) in both the primary and secondary enrichment cultures (Fig. 2A). With the pre-inoculation of *E. coli* O157:H7 to emulate different contamination scenarios, the overall degrees of *Clostridium sensu stricto 1* enrichment post-culture remained comparable to that of the contamination-free control groups, but no significant change in the abundance of the indigenous *Hafnia-Obesumbacterium* population was detected (Fig. 2). Instead, regardless of the initial O157:H7 inclusion levels (Fig. 2B and C), the abundance of *Escherichia-Shigella* bacteria in the culture was significantly increased by ∼7.5 log-fold after the primary enrichment step, with the secondary step further promoted the propagation of this bacterial genus to a total increment of ∼8.7 log-fold. Collectively, these findings not only echo the cultural isolation results (Table 2), but further reveal the non-competing co-selection of *Clostridium sensu stricto 1* bacteria, together with the *E. coli* O157:H7 target, from raw PBMA products under mTSB-based enrichment conditions. Additionally, the lack of significant *Hafnia-Obesumbacterium* enrichment in PBMA samples carrying *E. coli* O157: H7 clearly demonstrates the selective advantage given to the targeted bacterial pathogen (when present) over selected members of the sample's core microbiota (Table 1), which would otherwise have been selected for using this cultural method (Fig. 2A).

While co-enrichment of non-target microorganisms residing in food is not uncommon during selective enrichment − a notorious phenomenon that can be attributed to the off-target selectivity of method used and/or the overwhelming abundance of background bacteria relative to the intended target organism, the present finding on *Clostridium sensu stricto 1* enrichment associated with PBMA products appears to be a peculiar one. Clostridia are Gram-positive, anaerobic, rod-shaped bacteria capable of producing endospores, and can be ubiquitously found in different environments such as soil, water, plant, and the gut of human and animal (Dürre, 2014). Endospores represent a dormant/resting phase of *Clostridium* bacteria and are highly-resistant to heat, chemicals, desiccation, radiation, and even antibiotics (Dürre, 2014); thus can potentially persist in food and food-producing environment. Given that *Clostridium sensu stricto* consists of well over 100 species, encompassing both animal-associated pathogens and plant-associated saprophytes (Li et al., 2023), it is therefore tempting to speculate that the PBMA samples might harbour endospores formed by aerotolerant species of *Clostridium* that could be originated from the plant ingredients of this food product. During the selective enrichment process, and in an *E. coli* O157:H7-independent manner, the availability of nutrient/resources and the mesophilic conditions used might have triggered the germination of these endospores into vegetative cells that are metabolically-active and able to reproduce. As a result, the bacterial DNA that are also more easily extracted from the vegetative cells than from the hard-to-lyse, non-dividing endospores could potentially contributed to the increased 16S rRNA gene abundance as detected from the enrichment cultures. Regardless, further investigation is warranted to elucidate this phenomenon.

In regards to study limitations, the raw PBMA samples were acquired “as-is” from retailers and only six major brands available at the Canadian market were included in this study; thus the microbiota descriptions provided herein might not definitively represent the natural and *bona fide* bacterial content of this unconventional food commodity across different manufacturing processes and storage conditions. Additionally, samples were only subjected to a reduced equilibration period of 24 h following inoculation, potentially reducing lag times for STEC growth and leading to increased relative proportions in enrichment cultures. Note also that *E. coli* O157:H7 colonies were not confirmed as described in the MFHPB-10 method; however, given concordance of 16S data with presumptive *E. coli* O157:H7 enumeration, this was not deemed to be necessary for the purposes of this study. Moreover, the use of a short-read based sequencing methodology targeting only selected hypervariable regions of the 16S rRNA gene failed to discriminate closely-related taxa and was unable to offer (sub-) species-level taxonomic resolution, both of which are useful towards further characterizing the enrichment dynamics of *E. coli* O157:H7 and other background bacteria of raw PMBA products. Lastly, although there was no significant enrichment of any indigenous *Escherichia-Shigella* bacteria in the control samples (Fig. 2A–Table S4), it cannot be completely ruled out that these bacteria might have been co-enriched with the *E. coli* O157:H7 target in the contaminated samples (Fig. 2B and C) due to the aforementioned sequencing restrictions and/or potential mutualistic relationships that are yet to be proven.

## 4 Conclusions

In summary, the present study explored the microbiome of meat analogue products made with alternative protein sources (i.e. plant-based), to describe its overall bacterial composition and core microbiota components. Within the context of foodborne microbial hazard detection, the cultural dynamics of *E. coli* O157:H7 under the interference of the background bacteria carried by retail PBMA products was also characterized. Apart from demonstrating its proficiency in enriching for (and thereby facilitating the recovery of) foodborne *E. coli* O157:H7, results presented herein suggest that the mTSB-directed cultural enrichment strategy could select for off-target organisms such as *Clostridium sensu stricto 1* and *Hafnia-Obesumbacterium* bacteria from foods with potential safety concerns, including raw PBMA products. Finally, the highly selective enrichment of *E. coli* O157:H7 achieved using this specific cultural method suggests that the natural microbiota of raw PBMA products may lack the ability to out-compete *E. coli* O157:H7 under diagnostic settings, though this should not be generalized to all PBMA products due to limitations in variety of product brands and *E. coli* O157:H7 strains included in this study.

## Authors’ contributions

C.H-F.L. conceived, designed, and led the study. C.H-F.L. and S.C. designed and conducted the experiments. L.P.B. supported the bioinformatics analysis and sequencing data processing, and C.H-F.L. analyzed and interpreted the data. C.H-F.L. and C.D.C. contributed laboratory supplies and research resources. C.H-F.L. wrote the manuscript, and all authors contributed to and approved the final version of the manuscript.

## Declaration of competing interest

The authors declare that they have no known competing financial interests or personal relationships that could have appeared to influence the work reported in this paper.

## Figures and Tables

**Figure:**
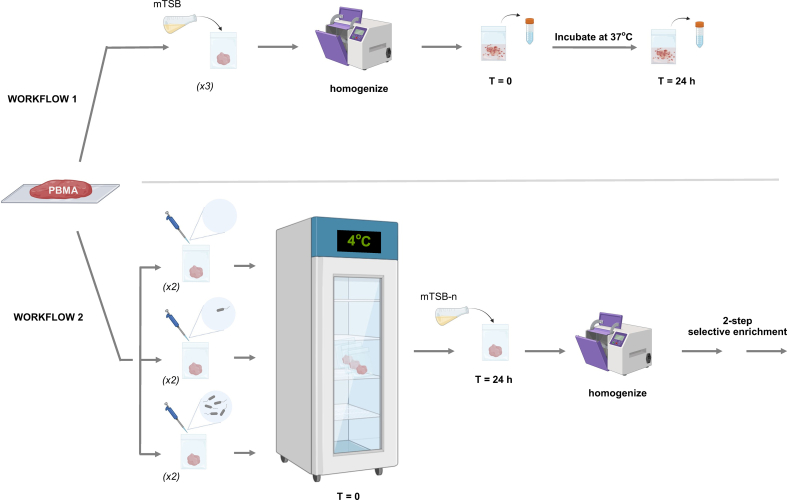
Fig. S1. Overview of sample processing workflows. Graphical illustration is created using biorender.com.

**Figure:**
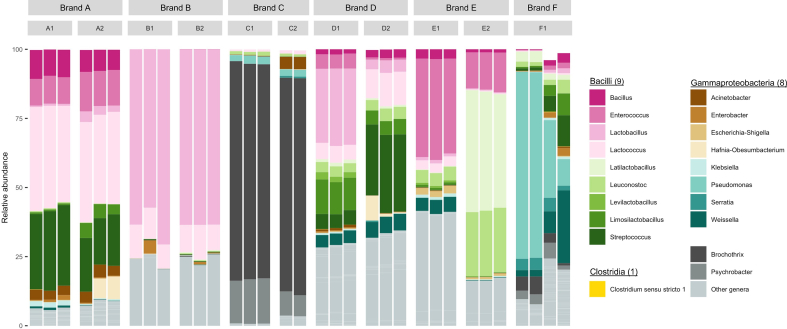
Fig. S2. Genus-level composition of plant-based meat analogues (PBMAs)-associated bacteria. Each stacked bar represents an independent sample replicate.

## Data Availability

Data will be made available on request.

## Appendix A Supplementary data

(Table S1. Inoculation levels of *E. coli* O157:H7 in artificially contaminated plant-based meat analogue samples/Table S2. PERMANOVA statistics for bacterial community dissimilarity/Table S3. Enumeration of total bacterial population associated with the plant-based meat analogue retail samples included in this study/Table S4. Relative abundance of presumed *Escherichia-*16S read for individual plant-based meat analogue samples and their corresponding enrichment cultures)
